# Deciphering Cadmium (Cd) Tolerance in Newly Isolated Bacterial Strain, *Ochrobactrum intermedium* BB12, and Its Role in Alleviation of Cd Stress in Spinach Plant (*Spinacia oleracea* L.)

**DOI:** 10.3389/fmicb.2021.758144

**Published:** 2022-01-24

**Authors:** S. Renu, Khan Mohd. Sarim, Dhananjaya Pratap Singh, Upasana Sahu, Manish S. Bhoyar, Asha Sahu, Baljeet Kaur, Amrita Gupta, Asit Mandal, Jyoti Kumar Thakur, Madhab C. Manna, Anil Kumar Saxena

**Affiliations:** ^1^ICAR-National Bureau of Agriculturally Important Microorganisms, Maunath Bhanjan, India; ^2^ICAR-Indian Institute of Vegetable Research, Varanasi, India; ^3^Intellectual Property Management Unit, National Innovation Foundation, Gandhinagar, India; ^4^ICAR-Indian Institute of Soil Sciences, Bhopal, India; ^5^ICAR-Indian Agricultural Research Institute, New Delhi, India

**Keywords:** cadmium, toxicity, bioremediation, *Ochrobactrum intermedium* BB12, spinach

## Abstract

A cadmium (Cd)–tolerant bacterium *Ochrobactrum intermedium* BB12 was isolated from sewage waste collected from the municipal sewage dumping site of Bhopal, India. The bacterium showed multiple heavy metal tolerance ability and had the highest minimum inhibitory concentration of 150 mg L^–1^ of Cd. Growth kinetics, biosorption, scanning electron microscopy (SEM), transmission electron microscopy (TEM), and Fourier transform infrared (FTIR) spectroscopy studies on BB12 in the presence of Cd suggested biosorption as primary mode of interaction. SEM and TEM studies revealed surface deposition of Cd. FTIR spectra indicated nitrogen atom in exopolysaccharides secreted by BB12 to be the main site for Cd attachment. The potential of BB12 to alleviate the impact of Cd toxicity in spinach plants (*Spinacia oleracea* L.) var. F1-MULAYAM grown in the soil containing Cd at 25, 50, and 75 mg kg^–1^ was evaluated. Without bacterial inoculation, plants showed delayed germination, decrease in the chlorophyll content, and stunted growth at 50 and 75 mg kg^–1^ Cd content. Bacterial inoculation, however, resulted in the early germination, increased chlorophyll, and increase in shoot (28.33%) and root fresh weight (72.60%) at 50 mg kg^–1^ of Cd concentration after 75 days of sowing. Due to bacterial inoculation, elevated proline accumulation and lowered down superoxide dismutase (SOD) enzyme activity was observed in the Cd-stressed plants. The isolate BB12 was capable of alleviating Cd from the soil by biosorption as evident from significant reduction in the uptake/translocation and bioaccumulation of Cd in bacteria itself and in the plant parts of treated spinach. Potential PGP prospects and heavy metal bioremediation capability of BB12 can make the environmental application of the organism a promising approach to reduce Cd toxicity in the crops grown in metal-contaminated soils.

## Introduction

Cadmium (Cd) is a transitional metal being excessively used in alloy plating, pigment production, and manufacturing of rechargeable batteries and phosphatic fertilizers (Muehe et al., 2013). Increased anthropogenic activities and geochemical processes in the last few decades have pounded the Cd level many folds in the aquatic and agricultural environment (Juan et al., 2018). In addition, the other most common route of Cd exposure for the human being is the consumption of contaminated food and deadly cigarette smoke (Rahimzadeh et al., 2017). Cd pollution has increased in various geographical regions at a steady rate over the last few decades, causing deadly threat to human health (Godt et al., 2006). The World Health Organization (WHO) has set the maximum permissible limit of Cd in drinking water (0.05 mg L^–1^) and in the plants (0.02 mg kg^–1^) (World Health Organization [WHO], 2008). Shi et al. (2019) comprehensively reported increased level of Cd in agricultural soils in China from 1981 to 2016. Average Cd content in rice plants grown at different locations was recorded to be 0.45 mg kg^–1^, a level exceeding the permissible limit of Cd in the food grains (Mu et al., 2019). Cd content in the food commodities and drinking water in many parts of world has been reported to be above the permissible limit as indicated in the reports from western Uttar Pradesh, India (Idrees et al., 2018); Bangladesh (Shaheen et al., 2016; Islam et al., 2018); Pakistan (Rehman et al., 2017); South Korea (Zhai et al., 2008); and China (Mu et al., 2019). High Cd content can inhibit the nutrient uptake from soil, restrict soil microbial population indirectly (Sanità Di Toppi and Gabbrielli, 1999), and affect the uptake of various growth-promoting elements, i.e., Ca, P, Mn, and K (Das et al., 1997). Cd in plants induces oxidative stress, destroys photosynthetic pigments, reduces stomata opening, and can severely damage the DNA (Wang et al., 2004). In animals and humans, evidence of Cd accumulation was found in the vital organs like kidney (Clemens et al., 2013). Hence, there is a dire need for the removal of such a life-threatening contaminant from the soil system, food chain, and human environment.

Physical and chemical methods, *viz.*, chemical precipitation, flocculation, membrane filtration, ultrafiltration, ion exchange, reverse osmosis, and electro-dialysis of Cd removal from the contaminated sites, are expensive, time consuming, and labor intensive (Yadav et al., 2021). Biological methods, on the other hand, are eco-friendly, safer for the soil and water systems, cost-effective, and are sustainable solutions. Microorganisms from different habitats, as biological systems, have been a premium choice for the heavy metal bioremediation (Renu et al., 2020; Shukla et al., 2020). The use of plant growth-promoting (PGP) bacteria for eco-friendly plant growth promotion in various crops has intensified in present time (Oleńska et al., 2020). Several mechanisms such as phosphate solubilization, nitrogen fixation, siderophore synthesis, and phytohormone production have already been documented (Kloepper et al., 1989; Glick, 1995; Patten and Glick, 1996; Glick et al., 1999). These microorganisms also possess multiple traits of bioremediation, biotransformation, biodecomposition, and biodegradation that may help to relieve the environment, soils, water, and plants from the toxicity of heavy metals and other contaminants (Hrynkiewicz and Baum, 2014; Ma et al., 2016). Metal-resistant PGP bacteria are reported to stimulate shoot and root growth of plants at enhanced levels of heavy metals and help plants to overcome metal stress (Igiri et al., 2018). To combat metal stress, microorganisms employ variety of mechanisms including biosorption on cell surface, immobilization/mobilization, extra/intracellular sequestration, complex formation with thiol-containing molecules, active efflux system, and conversion of highly toxic forms of compounds into less toxic (Gibbons et al., 2011). Biosorption has been considered as the environmentally and economically efficient process used by many researchers to revive environmental niches from heavy metal contamination (Kobya, 2004; Sarin and Pant, 2006; Saha and Orvig, 2010; Ayangbenro and Babalola, 2017).

Direct remediation of Cd by microbial system is less studied. This is because of the fact that Cd exists in the environment in only one state, i.e., +2. Thus, the direct transformation of Cd (like arsenic and chromium) is not possible. Biosorption has been reported as the primary and effective way of heavy metal removal by the microorganisms (Kanamarlapudi et al., 2018). Bioaccumulation of Cd in different pockets of cells has been demonstrated in *Burkholderia cepacia* (Zhang et al., 2019). Synergistic association of heavy metal–tolerant PGP bacteria with plants provides great opportunities for remediation potential of metal-contaminated habitats (Gururani et al., 2013; Babu et al., 2015; Gupta et al., 2018). In the present study, we have reported a Cd-tolerant bacterium isolated from the metal-contaminated site for its bioremediation potential and evaluated PGP ability on spinach. The study focuses on the ability of spinach plants to withstand Cd stress in the presence of the bacterium with PGP traits. We also reported in-depth mechanism of Cd tolerance in the bacterium and possible role of the isolate in alleviation of Cd toxicity in the spinach plants.

## Materials and Methods

### Sample Collection and Heavy Metal Analysis

Survey of various heavy metal–polluted sites and collection of metal-polluted bulk soils, rhizosphere soils of plants growing on metal-contaminated sites, and polluted water body samples was performed in Nagpur and Bhopal, India, as per the procedures mentioned by Leung et al. (2021) following the United State Environmental Protection Agency (USEPA) methods (Supplementary Table 1). Samples were collected in sterilized tight screw cap tubes and polyethylene bags, transported in an icebox, and stored in the laboratory at 4°C to avoid further oxidation/contamination. Collected samples were analyzed for heavy metal content using inductively coupled plasma (ICP) spectrometry as per the standard method (Krishna et al., 2009).

### Test Chemicals and Media

The stock solution of Cd (1,000 mg L^–1^) was prepared by dissolving Cd chloride (CdCl_2_) in Milli-Q water and filter sterilized through 22-μm filter (Axiva). Working test metal solution (20 mg L^–1^) was prepared by diluting the concentrated stock solution. All the glasswares were acid washed before use to avoid binding of metals. All the chemicals and culture media were of analytical grade and obtained from Sigma, Merck, and HiMedia.

### Isolation and Morphological Characterization of Bacteria

Various heavy metal–tolerant bacteria were isolated by serial dilution technique on Nutrient Agar (NA) media plates incubated at 37 ± 1°C for 24–48 h (Lima e Silva et al., 2012). Pure bacterial cultures were observed for characters like growth type and growth rate, and the bacterial colonies were subjected to microscopic observation using a light microscope (Olympus, Inc.). The observation on colony characteristics (color, texture, size, shape, margins, elevation, etc.) was recorded. Stereomicroscopic photographs of all the bacterial cultures were documented. Collection site, sample source, and Cd content in the samples are reported in Supplementary Table 1.

### Determination of Minimum Inhibitory Concentration

Minimum inhibitory concentration (MIC) of Cd for the bacterial isolates was determined following the method of Dey et al. (2016). Comparative MIC value of eight bacterial isolates for other heavy metals was also determined. Spot inoculation of 10 μl of 10^8^cells ml^–1^ bacterial suspension was performed on NA plates amended with Cd salt concentrations from 30 to 150 mg L^–1^. For other heavy metals, varied tested concentrations are mentioned in Supplementary Table 2. The results were recorded after 48 h of incubation at 28°C. The concentration of the metal that permitted growth and beyond which there was no growth was considered as the MIC of the metal against the tested isolate. Appropriate positive controls were kept without heavy metal content for the observation of growth of bacterial isolate. Out of all these isolates, the isolate BB12 was found most appropriate on trait basis to extend the study further.

### Mechanism of Cadmium Tolerance in Potential Bacterial Isolates

#### *In vitro* Growth Pattern and Biosorption Potential of Cadmium-Tolerant Bacterium

On the basis of MIC results, eight potential Cd-tolerant bacterial isolates, namely, BA3, BA4, BB4, BB12, BB13, BB14, BB15, and NR5, were selected for a batch culture study over the period of 10 days and were grown in the presence of 90 mg L^–1^ Cd in nutrient broth (NB) medium except BB15, which was grown in 40-mg Cd-amended NB medium. All the flasks were incubated on a rotary shaker (150 rpm), and optical density (OD) was measured at 600 nm every 2 h in spectrophotometer (Shimadzu, Japan). Growth was recorded against a reference. At 12, 24, 120, 168, and 240 h, 100-ml aliquot from each flask was withdrawn and pelleted by centrifugation at 6,000*g* for 10 min. Both pellet and supernatant were stored at 4°C and were analyzed for the presence of Cd through inductively coupled plasma atomic emission spectroscopy (ICP-AES).

#### Identification of Potential Cadmium-Tolerant Isolate BB12

Total genomic DNA was extracted using the CTAB method (Wilson, 2001). Primers pA (5′AGAGTTTGA TC CTGGCTCAG3′) and pH (5′AAGGAGGTGATCCAGCCG CA3′) were used for the amplification of 16S rDNA (Edwards et al., 1989). A 25-μl reaction mixture was prepared using 1.0 μl of 100-pM forward and reverse primers, deoxynucleoside triphosphate (DNTP) (200 nM), and 2 μl of 100-ng template. The amplification conditions were as follows: initial denaturation of 5 min at 94°C followed by 40 cycles of 40 s at 94°C, 40 s at 53°C, and 1 min and 30 s at 72°C, and a final extension period of 7 min at 72°C. The nucleotide sequences were dideoxy cycle sequenced with fluorescent terminators (Big Dye, Applied Biosystems) and run in 3130xl Applied Biosystems ABI prism automated DNA sequencer. Sequence alignment and comparison were performed using the multiple sequence alignment program CLUSTAL W (Thompson et al., 1994). The phylogenetic tree was reconstructed on the aligned datasets using neighbor joining (NJ) by Saitou and Nei’s (1987) method in MEGA 6 program (Tamura et al., 2013). Bootstrap analysis was performed as described by Felsenstein (1985) on 1,000 random samples taken from the multiple alignments. Partial 16S rRNA gene sequence of the bacterium was submitted to NCBI GenBank, and well-characterized strain was submitted to ICAR-National Agriculturally Important Microorganisms Culture Collection (NAIMCC), Mau, India.

#### Scanning Electron Microscopy and Fourier Transform Infrared Analysis of the Isolate BB12

The changes in cell surface morphology of the bacterial isolate were observed under scanning electron microscopy (SEM) and Fourier transform infrared (FTIR) as described by Sodhi et al. (2020). The isolate BB12 was raised in NB amended with 25 mg L^–1^ of Cd concentration. Cells were visualized under scanning electron microscope as described by Hou et al. (2015). Bacterial cells were harvested and fixed in 2.5% glutaraldehyde solution for 4 h at 4°C following washing thrice in 0.1 M phosphate buffer (pH 7.4). Post-fixation was done by treating samples with 1% osmium tetraoxide for 2 h at 4°C and washing thrice again for 15 min. Specimens were dehydrated using increasing concentrations (30, 50, 70, 90, and 100%) of ethanol for 30 min each time to evacuate water. Specimens were air-dried, mounted on aluminum stubs with carbon tape, and observed under scanning electron microscope. The infrared (IR) spectrum was obtained from FTIR spectrometer (Thermo Scientific) using potassium bromide (KBr) pellets (Gola et al., 2018). Bacterial cells (100 mg) were washed and freeze-dried, and 1-mg grounded biomass was mixed with 99 parts of KBr. This mixture was mounted on KBr to make it in to pellet form. FTIR analysis of freshly prepared bacteria-KBr pellet was performed in between wavelength of 400 to 4,000 cm^–1^.

#### Transmission Electron Microscopy Analysis of the Isolate BB12

For transmission electron microscopy (TEM) analysis, the isolate BB12 was grown (as in SEM analysis), and one drop of bacterial cells was placed on carbon-coated grid, stained with 2% urinyl acetate, and was drained with Whatman filter paper and viewed under TEM (TEM Jeol 1011, Japan) 80 KVA (Jain and Bhatt, 2014). Each treatment was prepared in triplicate.

#### Antibiotic Resistance and Plant Growth-Promoting Traits in the Isolate BB12

Antibiotic resistance/susceptibility of the isolate BB12 was ascertained against 20 antibiotics, *viz*., Amikacin (30 μg), amipicillin (10 μg), amoxicillin (10 μg), cefoperazone (75 μg), cefadroxil (30 μg), ceftazidime (30 μg), ceftriaxone (30 μg), chloramphenial (30 μg), ciprofloxacin (5 μg), cloxacillin (1 μg), co-trimoxazale (25 μg), erythromycin (15 μg), gentamicin (10 μg), nalidixic acid (10 μg), netillin (10 μg), nitrofurantain (300 μg), norfloxacin (10 μg), penicillin (10 units), tobramycin (10 μg), and (30 μg). The culture of BB12 was plated onto a nutrient agar plate, and an antibiotic disc (Icosa 002, HiMedia Pvt., Ltd.) was placed over it. Subsequently, the plates were incubated at 30°C for 24 h to observe the bacterial tolerance against different antibiotics by measuring zone diameter.

The isolate BB12 was also screened *in vitro* for various PGP traits such as siderophore production by the CAS agar method and Universal Chemical Assay (CAS) by Schwyn and Neilands (1987) and Milagres et al. (1999); ammonia production by Cappuccino and Sherman (2014); phosphate solubilization (Nautiyal, 1999); IAA production by Gordon and Weber (1951) and Loper and Schroth (1986); and K solubilization on modified Aleksandrov medium plates by the spot test method (Sindhu et al., 1999). The impact of 0, 25, 50, and 75 mg L^–1^ Cd was tested on PGP activity of the isolate BB12.

#### Evaluation of Cadmium Toxicity Alleviation Ability of BB12 in Spinach

The effect of varying concentrations of Cd on spinach plants was studied in a pot trial in complete randomized design (CRD). Sandy loam textured soil was collected from non-contaminated fields, air-dried, sieved through a 2-mm mesh, and homogenized. A total of 5.0 kg of air-dried sterilized soil was placed in each pot. Three concentrations (25, 50, and 75 mg kg^–1^) of Cd supplied in the form of CdCl_2_ were mixed with soil and kept for a week to stabilize the mixture. The soil without metal treatments served as a control. Seeds were placed on a paper towel and were allowed to germinate, and further seed biopriming was done by treating seeds with 0.1% carboxymethylcellulose and freshly grown culture of BB12 having cfu > 10^8^ml^–1^. Seeds were allowed to dry and sown in the pots containing soil having various concentrations of Cd. There were a total of four treatments, each having three replications. Pots were kept in the glasshouse and watered at regular intervals. In each pot, eight seeds were sown at a depth of 5 cm. After germination, five seedlings were maintained in each pot. Samples were withdrawn at different time intervals [i.e., 45 and 75 days after sowing (DAS)] and tested for physiological and biochemical attributes like chlorophyll (Chl) (Arnon, 1949), proline (Bates et al., 1973), and SOD (Beauchamp and Fridovich, 1971). To assess the impact of Cd content and Cd-tolerant bacteria on spinach, data on shoot length, shoot fresh and dry weight and root length, and root fresh and dry weight were recorded and were subjected to principal component analysis (PCA) and clustering analysis.

For estimation of Cd concentration, plants were harvested and roots and shoots were oven dried at 70°C for 48 h, powdered, and sieved through 2-mm mesh size. Residual Cd in dried shoot, root, and soil was estimated through ICP-OES. Translocation factor (TF) is the ability of plants to translocate heavy metal from root to shoot. Bioaccumulation factor (BAF) is expressed in terms of the ratio of Cd accumulated in shoot versus the content remained in the soil. TF and BAF were calculated using formulas given in Eqs 1, 2:


(1) Translocation⁢factor⁢(TF)=Cd⁢concentration⁢in⁢shoot/Cd⁢concentration⁢in⁢root

(2) Bioaccumulation⁢factor⁢(BAF)=Cd⁢concentration⁢in⁢shoot/Cd⁢concentration⁢in⁢soil

### Statistical Analysis

Unless otherwise stated, all the experiments were performed in triplicate. The data from the experiments were subjected to the Analysis of Variance (ANOVA) using SPSS Statistics V 20.0 with Duncan’s multiple range test (DMRT) at *p* < 0.05. Variation in the experimental data was expressed in terms of standard deviation (SD) and *p* < 0.05. PCA and cluster analysis was performed in PAST 4.03.

## Results

### Heavy Metals Content in Soil and Water Samples

Upon estimation, the content of majority of heavy metals was found above their permissible limit set by WHO and Central Pollution Control Board (CPCB), India. An alarming contamination level of Cd, i.e., 11.48 and 11.19 mg kg^–1^, was estimated in the sample collected from the Municipal Sewage Waste dumping site, Bhanpura, Bhopal (sample BB) and agricultural soil irrigated with Nag river water (sample MF1), respectively. Beside Cd contamination, the content of other heavy metals including Pb (1,716 mg kg^–1^), Cr (156.92 mg kg^–1^), and Co (58.98 mg kg^–1^) was also recorded in the bulk soil of heavy metal–contaminated site of Bhanpura, Bhopal (BB). Although the river of water of Nag contained low quantity of heavy metals except for Pb, the samples collected from the river irrigated soils of Nagpur, Maharashtra, India, showed high heavy metal content (Supplementary Table 1).

### Morphological Features of the Bacterial Isolates

For all the 45 bacteria isolated from the contaminated sites, the optimum growth temperature was found to be 37°C, and the optimum time ranged from 12 to 48 h. The colony color ranged from white to yellow to brown; the shape of the colony was mostly round with pointed at end; irregular and colony thread-like size ranged from 0.01 to 1 cm; surface smooth or rough and elevation was either flat or convex or raised.

### Heavy Metal Tolerance Using Minimum Inhibitory Concentration Test

Eight bacterial isolates exhibiting MIC for Cd in the range of 50–150 mg L^–1^ were selected for further studies (Supplementary Table 2). MIC of the isolates against eight other heavy metals, *viz*., Pb^+2^, Ni^+2^, Cr^+3^, Hg^+2^, Cu^+2^, Zn^+2^, Co^+2^, and As^+2^, was found to be in the range of 250–1,100 mg L^–1^ for As; 200–1,000 mg L^–1^ for Pb; 70–150 mg L^–1^ for Co; 200 mg L^–1^ for Cu; 100–500 mg L^–1^ of Cr; 100 mg L^–1^ for Hg; 100–150 mg L^–1^ for Ni; and 100–400 mg L^–1^ for Zn (Supplementary Table 2). Among all the isolates, BB12 was found to have highest MIC value (150 mg L^–1^) against Cd, and therefore, it was selected for further studies with this heavy metal.

### Identification of BB12 Isolate

**FIGURE 1:**
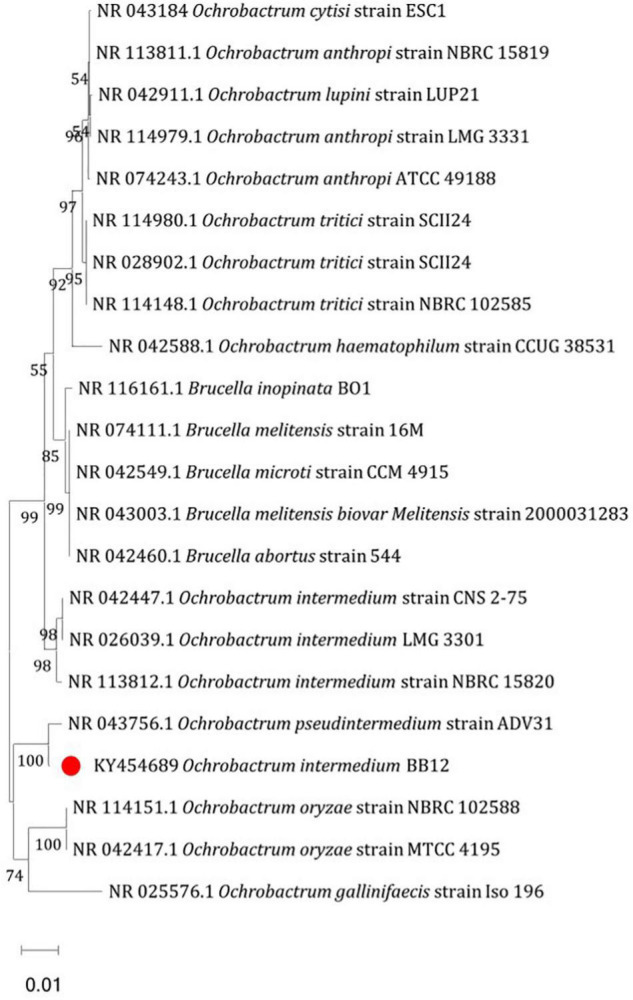
Neighbor-joining tree based on 16S rRNA sequence of heavy metal–tolerant bacteria *Ochrobactrum intermedium* BB12 by MEGA 6. The bars that corresponded to 0.01 substitutions per nucleotide position were indicative of scales of genetic distances. The numbers at branching nodes indicated the levels of bootstrap support (% of 1,000 replications); only those values above 50% are given.

On the basis of 16S rRNA gene sequence analysis, potential Cd-tolerating bacterium BB12 was identified as *Ochrobactrum intermedium* BB12 (Figure 1). The sequence was deposited in GenBank with accession number KY454689. The culture was submitted to National Depository ICAR-NAIMCC, Mau, India, with the accession number NAIMCC-B-02114.

### Bioremediation Character of Cadmium-Tolerant *Ochrobactrum intermedium* BB12

A biosorption study with the BB12 bacterial isolate was conducted by growing the bacterium in the Cd-containing media. With the help of ICP-AES analysis, it was possible to trace the pellet and supernatant for the Cd concentration at different time intervals of growth. Decreased Cd concentration in the supernatant but increased content in the corresponding cell pellet clearly indicated a possible mechanism of biosorption or bioaccumulation for Cd tolerance in the bacterium BB12. The highest level of Cd was observed in the pellets of BB12 (435.0 ± 1.00 mg kg^–1^) after 240 h of inoculation showing the biosorption potentials of these isolates (Supplementary Table 3). This prompted us to further evaluate the mechanism of biosorption in the bacterium.

### Scanning Electron Microscopy and Fourier Transform Infrared Analysis of *Ochrobactrum intermedium* BB12

**FIGURE 2:**
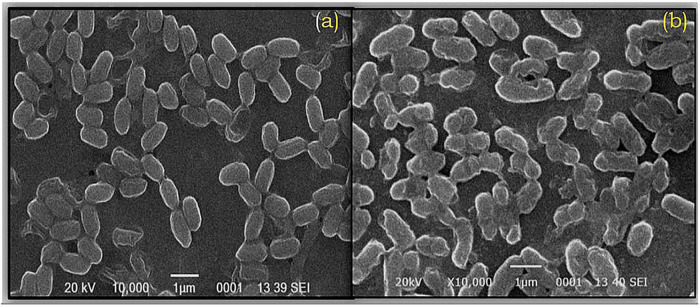
Scanning electron microscopy (SEM) of *Ochrobactrum intermedium* BB12 grown in **(a)** nutrient broth (control condition) and **(b)** nutrient broth amended with 25 mg L^–1^ Cd concentration (magnification × 10,000; 20 kV).

Scanning electron microscopy results indicated that the cells of *O. intermedium* BB12 without Cd were healthy with an average diameter of 1.48 ± 0.11 μm × 0.433 ± 0.01 μm. When grown with Cd-stressed condition, cell size was approximately 1.40 ± 0.11 μm × 0.414 ± 0.01 μm as observed under SEM. Cell surface deposition of Cd could be observed by the presence of a thick layer and distorted cell wall in the presence of Cd (Figures 2A,B).

**FIGURE 3:**
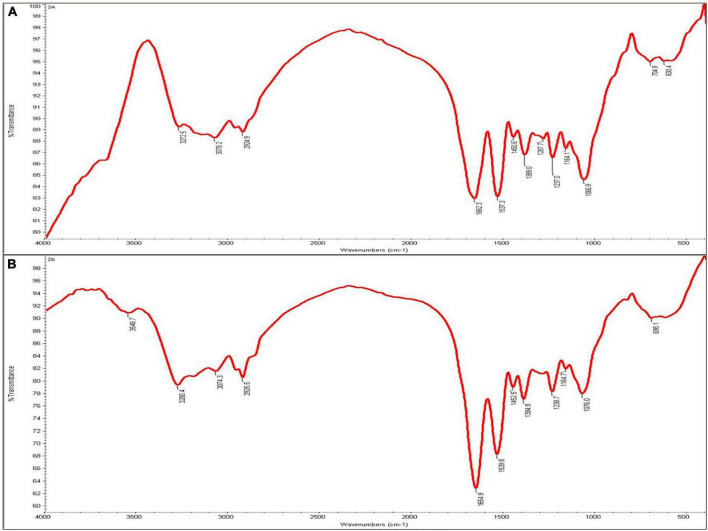
Infrared spectra of **(A)** *Ochrobactrum intermedium* BB12 grown in nutrient broth (control) and **(B)** media amended with 25 mg L^–1^ Cd concentration.

The interaction between bacterial cell wall and Cd was assessed by FTIR analysis. The FTIR spectrum was recorded from 400 to 4000 cm^–1^, which indicated that the Cd-treated *O. intermedium* BB12 revealed peaks at 1,662.3 and 1,537.3 cm^–1^, which were mainly attributed to the amide (I and II). The bands in non-treated cells had shifted to 1,654.9 and 1,539.8 cm^–1^, respectively. In Cd-treated cells, there was appearance of a peak at 3,548.7 cm^–1^ and shifting of 3,272.5, 3,078.2, and 2,924.9 to 3,280.4, 3,074.3, and 2,925.5 cm^–1^ that generally correspond to interaction with hydroxy and amino moieties of cell surface. Shifting of peaks at 1,450.6, 1,389.0, and 1,066.9 to 1,452.5, 1,394.9, and 1,076.0 cm^–1^, respectively, on exposure to Cd was also observed (Figures 3A,B).

### Transmission Electron Microscopy Analysis of *Ochrobactrum intermedium* BB12

**FIGURE 4:**
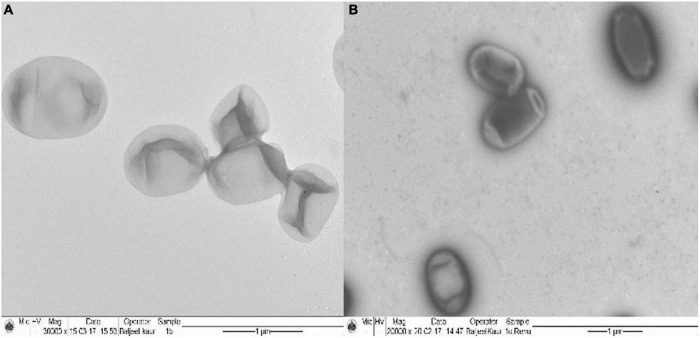
Transmission electron micrograph of **(A)** *Ochrobactrum intermedium* BB12 grown in **(A)** nutrient broth (control) (magnification, ×30,000) and **(B)** in media containing 25 mg L^–1^ Cd concentration (magnification, ×20,000).

*Ochrobactrum intermedium* colonies raised in the absence and presence of Cd (25 mg L^–1^) were subjected to the examination under TEM for the possible accumulation of Cd. Unstained bacterial preparation of in Cd control showed a clear homogenous cell cytoplasm with only a few tiny electron dense structures around the cell (Figures 4A,B). In contrast, the transmission electron micrograph of Cd-stressed bacterial cells exhibited opaque, electron dense area near the periphery of cells, clearly indicating the accumulation of Cd.

### Evaluation of Antibiotic Resistance and Plant Growth-Promoting Traits in *Ochrobactrum intermedium* BB12

*Ochrobactrum intermedium* BB12 was found to be resistant to amipicillin, amoxicillin, cefadroxil, ceftazidime, ceftriaxone, cloxacillin, nitrofurantoin, penicillin, and vancomycin and susceptible to amikacin, cefaperazone, chloramphenial, ciprofloxacin, co-trimoxazole, erythromycin, gentamicin, nalidixic acid, netillin, norfloxacin, and tobramycin (Supplementary Table 4).

**FIGURE 5:**
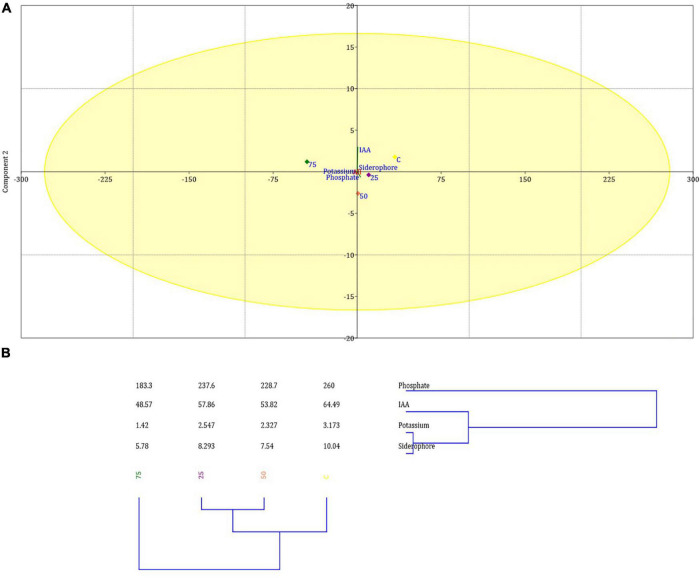
Principal component analysis **(A)** and clustering analysis **(B)** showing the impact of 25, 50, and 75 mg L^–1^ Cd concentration on various PGP traits of *O. intermedium* BB12 in the culture media. Mean of PCA-axis values ± SD (*n* = 3, *p* < 0.05).

*Ochrobactrum intermedium* BB12 was also screened *in vitro* for various PGP traits and was found positive for siderophore and IAA production and P and K solubilization (Supplementary Table 5). The relationship between test variables, *viz.*, indole acetic acid (IAA), siderophore, phosphate, and potassium against 0, 25, 50, and 75 mg kg^–1^ Cd content, was shown to be Cd concentration dependent as reflected in the PCA and clustering analysis of PGP traits (Figures 5A,B). Higher Cd concentration diminished the PGP attribute. The first two PCs have explained >80% variance of the data (Supplementary Table 6). It was observed that the first component contributed most in IAA, potassium, and siderophore production, whereas the second one contributed mostly in the P solubilization. The percent of PC1 was 99.645 followed by PC2 0.35422.

### Evaluation of *Ochrobactrum intermedium* BB12 on Alleviation of Cadmium Toxicity in Spinach

The exposure of various levels of Cd resulted in the growth retardation. Delay in the growth and development of Cd-stressed spinach plants was observed as compared to the control. All the seeds irrespective of various levels of Cd treatment germinated after 18 DAS, whereas in the control without *O. intermedium* BB12 treatment and without Cd stress, seeds germinated after 10 DAS. In bacteria-amended soil, the seed germination was recorded after 10–14 DAS. It was, therefore, speculated that *O. intermedium* helped in reducing the time of seed germination in the Cd-treated soil.

**FIGURE 6:**
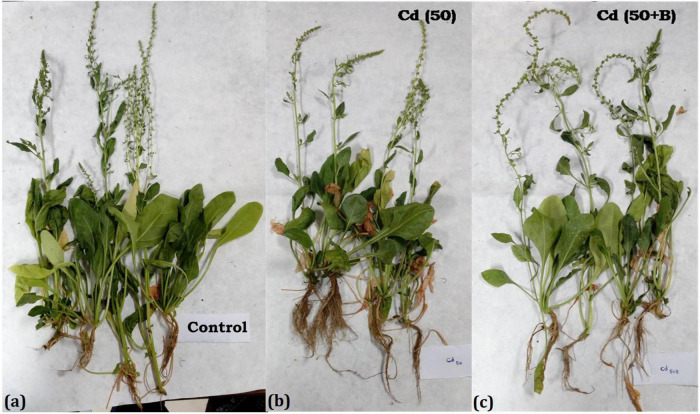
Growth of spinach in the soil treated with Cd and amended with the *Ochrobactrum intermedium* BB12 at 75 DAI. **(a)** Control: plants grown in the soil without any treatment; **(b)** Cd50: plants grown in soil containing 50 mg kg^–1^ Cd; and **(c)** Cd50 + B: plants grown in the soil containing 50 mg kg^–1^ Cd and inoculated with *O. intermedium* BB12.

**FIGURE 7:**
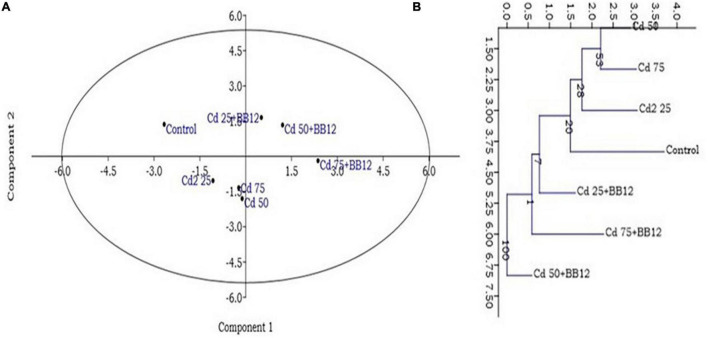
Principal component analysis (PCA) **(A)** and clustering analysis **(B)** showing the impact of 25, 50, and 75 mg kg^–1^ Cd concentration on the spinach plants grown in the soil containing Cd as compared to normal soils with no Cd concentration. Mean of PCA-axis values ± SD (*n* = 3, *p* < 0.05).

The treatment of 25 mg kg^–1^ of Cd in the soil significantly reduced the shoot length and shoot fresh weight but root length and root fresh weight were increased. On the other hand, root length and fresh weight in Cd-treated and bacterium-augmented plants were also higher than the control plants but less than only Cd-treated plants. There was decrease in shoot fresh weight, *viz.*, 9.03 ± 1.07, 7.27 ± 0.79, and 8.10 ± 1.15 gm in plants under 25, 50, and 75 mg kg^–1^ Cd content, respectively, in comparison to control (10.70 ± 0.90 gm). The application of bacteria increased the growth parameters like the number of leaves, root and shoot length, and fresh and dry weight of root and shoot observed at different days of sowing at different concentrations of Cd as compared to plants growing in Cd stress without bacterial treatment (Table 1). The presence of bulgy roots exhibits the ability of plants to counter heavy metal stress in bacteria-augmented plants under heavy metal stressed plants (Figure 6). In comparison to the plants grown in normal soil (control) (Figure 6A), those exposed to 50 mg kg^–1^ Cd concentration were reduced drastically in growth, but when the soil containing the same Cd concentration was inoculated with the bacteria, plant growth showed significant revival (Figure 6C). The PCA analysis suggested that Cd treatment (25, 50, and 75 mg kg^–1^) severely affected the growth parameters of plants (Figure 7A). For the observed impact of BB12 on the reduction of Cd uptake and physiological growth of spinach at 0, 25, 50, and 75 mg kg^–1^ of Cd, PC1 showed the % variance of 90.97% followed by PC2 8.8299 (Supplementary Table 7). It was observed that soil treatment with the bacteria BB12 exhibited improved plant growth against respective Cd concentrations. Cd25 + BB12 and Cd50 + BB12 treatments performed well although not much significant growth was observed in Cd75 + BB12 treatment, possibly because high concentration of metal restricted the growth. Further cluster analysis (Figure 7B) also strengthened these observations and suggested that reduced growth parameters are proportional to Cd concentration in the soil.

**TABLE 1:** Effect of *Ochrobactrum intermedium* BB12 on growth parameters of spinach plants under 25, 50, and 75 mg kg^–1^ Cd content at 45 and 75 days after sowing (DAS).

Growth parameters	DAS	T1	T2	T3	T4	T5	T6	T7
Number of leaves	45	3.67 ± 0.2^a^	3.67 ± 0.2*^a^*	4.00 ± 0.2*^a^*	3.33 ± 0.2*^a^*	4.00 ± 0.0*^a^*	3.33 ± 0.2*^a^*	3.67 ± 0.23*^a^*
	75	8.00 ± 1.00*^ab^*	6.67 ± 0.33*^b^*	9.67 ± 0.88*^a^*	8.00 ± 1.00*^ab^*	10.00 ± 0.58*^a^*	7.00 ± 0.33*^b^*	10.00 ± 0.67*^a^*
Root length (cm)	45	5.33 ± 0.88*^d^*	6.33 ± 0.88*^d^*	7.33 ± 0.33*^c^*	8.33 ± 0.33*^bc^*	11.33 ± 0.33*^a^*	9.67 ± 0.88*^ab^*	10.33 ± 0.88*^ab^*
	75	6.67 ± 0.67*^ab^*	10.33 ± 0.33*^c^*	12.00 ± 1.15*^a^*	10.67 ± 1.67*^bc^*	10.00 ± 0.58*^ab^*	11.00 ± 1.00*^bc^*	8.83 ± 0.93*^ab^*
Shoot length (cm)	45	14.33 ± 1.18*^abc^*	15.67 ± 1.16*^c^*	18.33 ± 1.19*^a^*	15.67 ± 1.19*^b^*	19.00 ± 1.28*^a^*	15.00 ± 1.20*^b^*	18.00 ± 1.29*^a^*
	75	47.33 ± 0.67*^a^*	36.33 ± 0.88*^c^*	43.33 ± 0.88*^b^*	32.67 ± 0.33*^d^*	47.00 ± 1.00*^a^*	35.33 ± 0.88*^c^*	36.67 ± 0.88*^c^*
Root fresh weight (g)	45	0.30 ± 0.06*^c^*	0.36 ± 0.06b*^c^*	0.41 ± 0.01*^bc^*	0.38 ± 0.04*^bc^*	0.51 ± 0.08*^ab^*	0.53 ± 0.03*^ab^*	0.62 ± 0.07*^a^*
	75	0.31 ± 0.4*^c^*	0.72 ± 0.16*^ab^*	1.01 ± 0.24*^a^*	0.73 ± 0.09*^ab^*	1.26 ± 0.23*^a^*	0.86 ± 0.08*^ab^*	1.13 ± 0.15*^a^*
Shoot fresh weight (g)	45	6.17 ± 0.50a^3^	6.27 ± 1.63^*a*2^	6.37 ± 0.79^*a*1^	4.60 ± 0.06^*a*6^	4.60 ± 0.52^*a*5^	4.20 ± 1.00^*a*7^	5.70 ± 0.21^*a*4^
	75	10.70 ± 0.90*^a^*	9.03 ± 1.07*^bc^*	9.47 ± 0.77*^ab^*	7.27 ± 0.79*^bc^*	9.33 ± 0.33*^abc^*	8.10 ± 1.15abc	6.67 ± 0.13*^c^*
Root dry weight (g)	45	0.23 ± 0.05*^b^*	0.25 ± 0.04*^b^*	0.32 ± 0.03*^ab^*	0.30 ± 0.09*^ab^*	0.38 ± 0.09*^ab^*	0.31 ± 0.03*^ab^*	0.46 ± 0.17*^a^*
	75	0.20 ± 0.02*^d^*	0.31 ± 0.12*^cd^*	0.52 ± 0.23*^bcd^*	0.50 ± 0.18cd	0.86 ± 0.27*^d^*	0.61 ± 0.16*^abc^*	0.84 ± 0.17*^ab^*
Shoot dry weight (g)	45	2.13 ± 0.58*^c^*	3.67 ± 0.25*^ab^*	4.07 ± 0.30*^a^*	3.60 ± 0.20*^ab^*	4.10 ± 0.10*^a^*	3.37 ± 0.20*^b^*	4.00 ± 0.20*^a^*
	75	2.67 ± 0.31*^bc^*	3.37 ± 0.84*^ab^*	3.63 ± 0.76*^ab^*	2.73 ± 0.23*^abc^*	3.13 ± 0.50*^ab^*	2.00 ± 0.66*^c^*	3.77 ± 0.31*^a^*

*Values are the means (±SD) of three replicates; within the same row with different letters, values are significantly different at p ≤ 0.05 in DMRT.*

*SD, standard deviation.*

*“a” stands for highest variation, followed by “b” and lowest by “d” from the experimental control; T1, control without Cd and Ochrobactrum intermedium BB12; T2, T4, and T6, Cd control at 25, 50, and 75 mg kg^–1^ Cd, respectively, without O. intermedium BB12 inoculation; T3, T5, and T7, O. intermedium BB12-treated plants under 25, 50, and 75 mg kg^–1^ Cd stress, respectively.*

The physiology of spinach plant was also altered under Cd stress condition. Chl content revealed the changes in the amount of pigment in expanding leaves at different concentrations of Cd. Chl-a concentration was significantly lower than that of Chl-b in plants. The pigment content in plants showed almost linear decrease in response to increase in Cd concentration in the soil. At 75 DAS, total Chl content (74.36 ± 3.16, 89.91 ± 3.55, and 70.54 ± 2.91 μg g^–1^) of plants under various levels of Cd stress (25, 50, and 75 mg kg^–1^, respectively) was found to be lower and in decreasing order of Cd content in comparison to control (88.31 ± 4.57 μg g^–1^). On the other hand, the total Chl content of 77.17 ± 4.43, 93.23 ± 4.97, and 78.97 ± 3.53 μg g^–1^ in the plants grown in the soil containing 25, 50, and 75 mg kg^–1^ Cd content, respectively, with bacterial inoculation indicated reduced impact of the Cd stress (Supplementary Table 8). Proline content was found to increase with increase in the level of Cd content, whereas bacteria treatment in the soil lowered down the proline content in Cd-stressed plants. At 45 DAS, proline content in the control plants (without Cd) was recorded as 0.63 ± 0.04 μmole g^–1^, which increased with the increase in Cd content (1.44 ± 0.27, 1.80 ± 0.05, and 1.89 ± 0.27 μmole g^–1^). In bacteria-amended plants, proline content was lowered down to 0.76 ± 0.02, 0.92 ± 0.04, and 1.09 ± 0.23 μmole g^–1^ at 45 DAS at 25, 50, and 75 mg kg^–1^, respectively (Supplementary Table 8). Soil inoculation with the bacteria lowered down the proline content in the plants as compared to those growing in the soil with Cd only. A similar trend was observed in the case of SOD enzyme production also.

**FIGURE 8:**
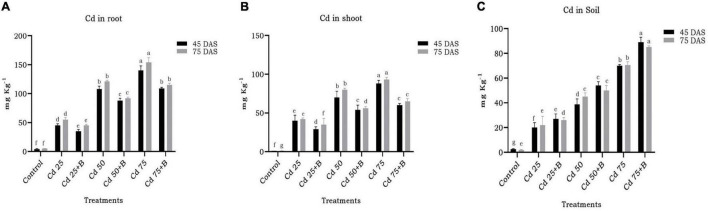
Effect of the application of *Ochrobactrum intermedium* BB12 on the uptake of Cd by the root **(A)** and shoot **(B)** of spinach plants grown in the soil containing 25 (Cd25), 50 (Cd50), and 75 (Cd75) mg kg^–1^ metal concentration at 45 and 75 days of sowing (DAS). Cd content in the soil measured at the same time interval is shown in panel **(C)**. B denotes the treatment containing bacteria *O. intermedium*. Values are the means (±SD) of three replicates; within the same column with different letters, values are significantly different at *p* ≤ 0.05 in DMRT. SD, standard deviation. “a” stands for highest variation, followed by “b” and lowest by “f” from the experimental control.

Biopriming of spinach seeds with *O. intermedium* BB12 led to statistically significant reduction in the uptake of Cd in different plant parts (Figure 7). In general, there was more accumulation of Cd in roots than shoots (Figure 8). In BB12-treated plant at 45 DAS, there was 22.22, 18.51, and 22.14% reduction, and, at 75 DAS, there was 18.18, 23.96, and 25.32% reduction of Cd uptake in roots in plants grown in soil amended with 25, 50, and 75 mg kg^–1^ Cd, respectively, as compared to without BB12-treated plants. Similarly, at 45 DAS, there was 27.5, 22.85, and 31.81% reduction, and, at 75 DAS, there was 16.66, 30, and 30.10% reduction of Cd uptake in shoots in the plants grown in soil amended with 25, 50, and 75 mg kg^–1^ Cd, respectively. Cd accumulation was 35, 39.53, and 27.14% more at 45 DAS and 18.18, 11.11, and 13.21% more at 75 DAS in 25, 50, and 75 mg kg^–1^ Cd-amended soil, indicating direct support of bacterial biopriming for the plants.

**FIGURE 9:**
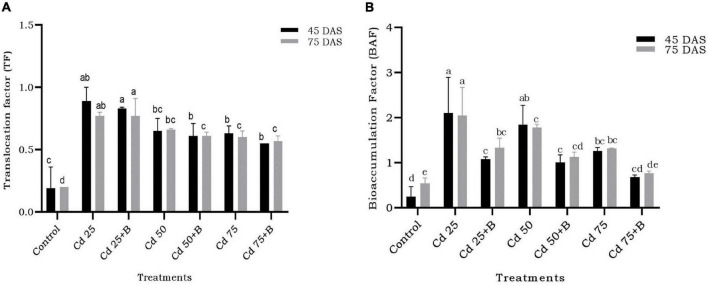
Effect of the application of *Ochrobactrum intermedium* BB12 on **(A)** translocation factor and **(B)** bioaccumulation factor of Cd by spinach plants grown in the soil containing 25 (Cd25), 50 (Cd50), and 75 (Cd75) mg kg^–1^ Cd content at 45 and 75 DAS. Values are the means (±SD) of three replicates; within the same column with different letters, values are significantly different at *p* ≤ 0.05 in DMRT. SD, standard deviation. “a” stands for highest variation, followed by “b” and lowest by “e” from the experimental control.

Translocation of Cd from root to shoot decreased due to bacterial inoculation but only at limited level. The effect of inoculation of BB12 was significant at 50 and 75 mg kg^–1^ Cd in reducing the translocation in comparison to the plants with Cd treatment only. The observed effect at 45 and 75 DAS was almost the same, thus indicating no impact of the time interval in translocation (Figure 9A). Bacterial inoculation resulted in bioaccumulation of Cd in the soil in which bacteria had significant effect at 50 and 75 mg kg^–1^ Cd in the soil at 45 and 75 DAS. Even at the higher dose of 75 mg kg^–1^ Cd, BB12 influenced Cd bioaccumulation in the soil to a greater extent (Figure 9B).

### *Ochrobactrum intermedium* BB12 Population in the Rhizospheres

Bacterial population in the rhizosphere of spinach was tested as CFU count at 35, 45, and 75 days of inoculation. At 75 days, the CFU count of BB12 was 4.3 × 10^5^, 4.0 × 10^5^, and 3.4 × 10^5^cfu g^–1^ rhizosphere soil, respectively, in 25, 50, and 75 mg kg^–1^ Cd-amended soil, indicating relatively lesser effect at different levels of Cd content in the soil on bacterial population.

## Discussion

Presence of heavy metals in the soils and high accumulation in plant parts resulted in retarded growth of several crop plants (Yadav, 2010; Chibuike and Obiora, 2014). Microorganisms tolerant to stress due to heavy metals can bioremediate soils with metal contamination (Ojuederie and Babalola, 2017). Similarly, their inoculation in the rhizosphere not only can help in alleviating the effect of metal stress in plants but also can support growth and development to a greater extent (Rajkumar et al., 2010). We have identified a bacterium having various PGP traits like siderophore and phytohormone production and P solubilization to hold Cd tolerance potential. The bacterial isolate BB12 obtained from a heavy metal–contaminated site in India was identified as *O. intermedium.* Although the species of the genus *Ochrobactrum* in general has been reported as opportunistic human pathogens of low virulence in humans (Ryan and Pembroke, 2020) and the species *O. intermedium* was implicated in a single case of liver infection (Wisplinghoff, 2017), these soil organisms possess multiple traits too. Several species have been investigated for their potential as biodegrader of xenobiotic compounds and detoxifier of heavy metals in a variety of environmental conditions (El-Sayed et al., 2003; Sultan and Hasnain, 2007). The bacterium has also been characterized to possess plant growth promotional traits and biofertilizer potential (Sharma et al., 2016). We have examined multiple traits of agricultural importance of the isolate BB12, which showed promise not only in plant growth promotion but also in the bioremediation of the soils contaminated with the heavy metals. All these multiple traits along with the ability to withstand high concentrations of Cd metal in the soils owe ecological stability to the organism to stand in the unfavorable conditions as well, thereby offering plants better protection against metal stresses. Besides Cd, the identified isolate has also shown tolerance against arsenic, chromium, cobalt, lead, and nickel, thereby indicating multi-metal tolerance and that also up to different limits of high concentration. Such organisms could be a better choice in the search of microbial inoculants as bioremediaters of metal-contaminated environmental sites.

Batch culture studies with tolerant strains inoculated in Cd-amended broth over a period of 10 days revealed the differential accumulation of Cd in the cell pellet and the supernatant. In general, the higher Cd concentration in the bacterial cell pellets as compared to that in the culture supernatant suggested that the mechanism of tolerance may either be the adsorption of Cd on cell surface or the bioaccumulation. Several studies have reported biosorption and intracellular accumulation as the two primary mechanisms of heavy metal tolerance in bacteria (Lu et al., 2006; Rajesh et al., 2014; Hou et al., 2015). The co-existence of heavy metal and antibiotic resistance in bacteria has been explained (Chen et al., 2019). Kaur et al. (2018) described that increased Cd concentration altered the susceptibility of bacterium against ampicillin, cefizoxime, chloramphenicol, and ciprofloxacin in *Salmonella enterica* and caused simultaneous morphological, biochemical, and physiological changes. Similar observation was also reported by Nath et al. (2019) who isolated heavy metal–tolerant bacteria from the industrial region with resistance to penicillin, ampicillin, vancomycin, co-trimoxazole, etc. Therefore, our observation on the simultaneous tolerance against Cd and resistance against several antibiotics in BB12 is in accordance with the earlier reports.

Scanning electron microscopy studies on *O. intermedium* revealed the distortions and changes in the cell morphology with bulging of the cell surface. The bulging could be due to high exopolysaccharide production under Cd stress as compared to control. Sultan et al. (2008) reported that the cell surfaces of cultures treated with Cd chloride tended to be rough, suggesting that the cell increased its surface to improve the interaction of toxic substances with the cell surface. El-Helow et al. (2000) and Sinha and Mukherjee (2009) demonstrated that *Bacillus* sp. contains cell wall components such as epoxypolysaccharides (EPS), teichoic and teichuronic acids or phospholipid layers, and functional groups can be responsible for the heavy metal bioremediation through the secretion of extracellular substances. Zhang et al. (2019) reported that, in *B. cepacia* GYP1, although there was distortion in the cell structure due to heavy metal toxicity, the integrity of cells remained intact. Similar to our observation, the authors have also emphasized that the accumulation of Cd was mainly on the outer membrane surface at the beginning, whereas the intracellular Cd intake increased and held stable after 2 days. Afterward, the increased amount of Cd was mainly located extracellular and was related to the secreted EPS. Our results on TEM of *O. intermedium* could reveal the intracellular sites of Cd accumulation. High density of electron was observed in periplasmic space of the cells, whereas some deposition was also seen in the cytosol and toward cell envelop. This has indicated that the Cd was also deposited within the bacterial cells. Intracellular deposition of Cd by TEM in various bacterial strains has already been reported (Vicentin et al., 2018; Zhang et al., 2019). Therefore, both the SEM and the TEM results strongly suggested that the bacteria possess the capability to withhold the Cd metal as extracellular deposition on its cell surface, and it can also deposit the metal in intracellular parts in the cytosolic region.

Fourier transform infrared analysis of cell surface provides a deep insight of functional groups on bacterial cell surface involved in the metal bacteria interaction. Negatively charged groups tend to bind positively charged heavy metal ion and *vice versa.* FTIR results showed that -COOH is the most active functional group involved in the cell–metal interaction. This was also previously reported by Pradhan and Sandle (1999) and Rajandas et al. (2012). The region from 3,700 to 3,300 cm^–1^ in IR spectra is characteristic of O-H and N-H stretching vibrations and 3,300–3,000 cm^–1^ of C-H stretching vibrations of C≡C, C=C, and Ar-H. The region from 3,000 to 2,700 cm^–1^ is dominated by the C-H stretching vibrations of -CH_3_, >CH_2_, CH, and CHO functional groups, respectively (Dumas and Miller, 2003; Guo and Zhang, 2004). In FTIR spectra of *O. intermedium* BB12, there was appearance of peak at 3,548.7 cm^–1^ and shifting of 3,272.5, 3,078.2, and 2,924.9 to 3,280.4, 3,074.3, and 2,925 in Cd-treated bacterial cells. Such shiftings could be attributed to O-H, N-H, and C-H stretching and thus indicated participation of hydroxyl and amino group in Cd interaction. It is interesting to note that –OH being negatively charged ion can have electrostatic interaction with the Cd ion easily. Shifting of peaks at 1,662.3 and 1,537.3 cm^–1^ to 1,654.9 and 1,539.8^–1^cm, respectively, in Cd-stressed bacterial cells suggested the involvement of amino group in Cd^2+^ attachment. The region from 1,600 to 1,500 cm^–1^ is specific for amide-II bands, which is due to N-H bending vibrations (Fischer et al., 2006). The region between 1,700 and 1,600 cm^–1^ is specific for amide-I bands (Dumas and Miller, 2003), which is mainly due to C=O stretching vibrations of the peptide bond (Backmann et al., 1996). Similar results were also obtained with Cd-resistant *S. enterica* 43C, in which chemical interaction with Cd due to amide group of the bacterium was inferred due to the shift in peaks at 1,635 and 1,552 cm^–1^ (Khan et al., 2016).

The intensity and stretching of the peak clearly demonstrated the alteration in the functional groups associated with the cell surface and strongly indicated molecular bonding of the metal ion at the surface. This could be one of the strong possible mechanisms of Cd removal from the culture media, in which the bacterium was grown and caused less concentration of Cd in the supernatant and high concentration in the cell pellets. On the other hand, shifting of any peak in IR spectra also informs about the change in hybridization state of molecular bonding. There was also shifting of peaks at 1,450.6, 1,389.0, and 1,066.9 to 1,452.5, 1,394.9, and 1,076.0, respectively. The bands in the 1,500–1,200 cm^–1^ region arise mainly from the C-H bending vibrations of CH_3_, CH_2_, and CH functional groups. The region from 1200 to 900 cm^–1^ is mainly dominated by a sequence of bands due to C-O, C-C, C-O-C, and C-O-P stretching vibrations of polysaccharides (Wolkers et al., 2004; Yee et al., 2004) as well as CH_3_ and CH_2_ rocking modes by Wolpert and Hellwig (2006). In our study, shifting of peaks in the above regions can also be attributed to the binding of Cd to polysaccharides, either associated with the outer membrane or excreted EPS on the surface, as has been suggested by Alessi et al. (2014). Zhang et al. (2019) have reported organic functional groups, such as carboxyl, amino, and phosphoryl groups on cell wall or extracellular polymeric substances to be involved in the removal of Cd by complexation with *B. cepacia* GYP1. It was also observed that, in case of both strains, the percentage transmittance was lesser in Cd-treated bacteria than in control. According to Chakravarty and Banerjee (2012) due to the presence of Cd, stretching of bonds occurred to a lesser degree, resulting in reduction in their percentage transmittance. Similar observations were also recorded in the interaction of *S. enterica* 43C with Cd (Khan et al., 2016). Therefore, our observations are very well in accordance with those reported earlier.

The above findings of the laboratory suggested that Cd-tolerant PGP bacterium *O. intermedium* BB12 may influence the mobilization of Cd in the soil and could be a suitable choice for bioremediation purposes. To validate the effects of BB12 inoculation in spinach under varying concentrations of Cd, pot experiments were designed with the soils containing different content of Cd. Spinach is a well-known hyperaccumulator of various heavy metals including Cd (Alia et al., 2015). The Cd stress affected all studied plant growth attributes, which were improved due to the inoculation with BB12. These observations were parallel to the existing report on the PGP attributes of the genus *Ochrobactrum* (Sharma et al., 2016). Even under Cd treatment conditions, the observed improvement in the growth and development of plants may cumulatively be attributed to the PGP traits like siderophore and phytohormone production and P solubilization of the bacteria. Alternatively, the observed effect on plant growth may be linked with the ability of the bacteria to remove Cd from the plant rhizosphere, thereby minimizing the exposure of roots with the heavy metal. This may also be resulted due to the ability of bacterial association to induce intrinsic defense mechanisms in the plants against the metal to tolerate toxicity effects of the Cd. Several workers have studied the heavy metal toxicity alleviation using PGP bacteria possessing traits like P solubilization, nitrogen fixation, siderophore, and phytohormone production (Tiwari and Lata, 2018; Caracciolo and Terenzi, 2021). These attributes have stimulated shoot and root growth of plants in the presence of enhanced levels of heavy metal and thus helped them in overcoming metal stress. The identified isolate BB12, besides having Cd tolerance, was also found to be a phosphate solubilizer and siderophore and phytohormone producer, and these are the agriculturally important traits for any microorganism to support plant growth and development in the field conditions. It has been reported that contamination of soil with heavy metal results in iron deficiency in different plant species (Mishra and Kar, 1974; Römheld and Marschner, 1986; Wallace et al., 1992; Ma and Nomoto, 1993; Burd et al., 2000; Gupta et al., 2018; El-Meihy et al., 2019) and causes inhibition of both chloroplast development and Chl biosynthesis, resulting in chlorosis in plants (Imsande, 1998). Microbial siderophore complexes with iron that can be taken up by plants help in iron acquisition (Reid et al., 1986; Bar-Ness et al., 1991; Wang et al., 1993). Hence, siderophore-producing bacteria could be a good choice for growing crops in the metal-contaminated soils to prevent plants from becoming chlorotic due to heavy metal impact. We also reported reduction in the Chl production in plants under Cd stress, but again, it was improved due to the application of bacterial inoculation.

Physiological parameters based on defense-related biomolecules and antioxidant enzymes were also found to affect plant growth and development during metal stress (Hasanuzzaman et al., 2020). In plants, stress induces production of reactive oxygen species (ROS), which are even more toxic to plant cells, and, for such reasons, bacteria accumulate stress-induced compounds like proline and antioxidant enzymes in the cells to rapidly reduce the load due to stresses (Sun et al., 2018). Proline serves as a plant-friendly metabolic osmolyte and does not sequester the contaminants but performs major functions to lower down the ROS production and restrict the role of GSH (Sharma et al., 2012). Similarly, during environmental stresses, ROS production increases causing high oxidative damage, which is controlled by these antioxidant enzymes like superoxide dismutase (SOD), which converts superoxide into H_2_O_2_. Altered activities of SOD have been reported as indicators of Cd stress in different plants (Foroozesh et al., 2012; Li et al., 2013). Increasing concentrations of Cd increased the proline production and SOD activity in spinach. However, in the presence of BB12, both proline production and SOD activity were decreased. This may be speculated due to the ability of BB12 to reduce the exposure of Cd stress to the plants. Reduced uptake of Cd was observed due to the inoculation of BB12. TF of bacteria-amended plant was found <1, thus indicating the low translocation. BAF is also greatly reduced in plants amended with Cd-tolerant bacteria. With the results, we realize that, owing to the Cd biosorption by the BB12 as indicated in *in vitro* tests, the minimized exposure of plant rhizosphere for the uptake of Cd up to such a level that could become toxic for plants has been restricted. In this way, the bacterium *O. intermedium* performed the role of microbial bioremediation to help spinach withstand with the metal stress. The study presents possibility of utilizing multi-trait microbial species like *O. intermedium* as inoculants for ensuring plant growth promotion in soils contaminated with heavy metals. Although, the occasional pathogenicity of *O. intermedium* may restrict its wide-scale direct application in the field conditions as bioremediator-cum-biofertilizer agent, as can be recommended for the bacteria with similar level agriculturally important traits, its high biosorption capability can be utilized for contained environmental applications to remediate highly heavy metal–contaminated sites. Alternatively, the immobilized cells of the bacteria BB12 on various low cost biosorbents can be applied for Cd remediation of contaminated sites and/or seed biopriming for supporting physiological growth of the plants as the ways of applications in industrial and agricultural fields.

## Data Availability Statement

The datasets presented in this study can be found in online repositories. The names of the repository/repositories and accession number(s) can be found in the article/Supplementary Material.

## Author Contributions

SR and ASx conceived and designed the study. KS, MB, US, AG, and SR performed laboratory and pot experiments, SEM, and FTIR. BK performed TEM. ASh, AM, JT, and MM conducted Cd analysis of *in vitro* and pot experiment samples. DS and KS analyzed the data. SR, KS, DS, and ASx drafted the manuscript. All authors commented on the manuscript and made suggestions.

## Conflict of Interest

The authors declare that the research was conducted in the absence of any commercial or financial relationships that could be construed as a potential conflict of interest.

## Publisher’s Note

All claims expressed in this article are solely those of the authors and do not necessarily represent those of their affiliated organizations, or those of the publisher, the editors and the reviewers. Any product that may be evaluated in this article, or claim that may be made by its manufacturer, is not guaranteed or endorsed by the publisher.
