# Supplementary material for: Deciphering Cadmium (Cd) Tolerance in Newly Isolated Bacterial Strain, Ochrobactrum intermedium BB12, and Its Role in Alleviation of Cd Stress in Spinach Plant (Spinacia oleracea L.)
Source: Front Microbiol. 2022 Jan 24;12:758144. doi: 10.3389/fmicb.2021.758144 (PMC8819065; doi:10.3389/fmicb.2021.758144)
Supplement: Supplementary file 1 [file Data_Sheet_1.docx]

Supplementary Materials

**Supplementary Information Table S1:** Bacterial isolates obtained from the samples of heavy metal polluted sites (metal-polluted water-bodyand bulk soil) along with heavy metal content by ICP-OES

| **Sample name** | **Sample** | **Location** | **Coordinates** | **Morphotypes** (numbers) | **Heavy metals content in environmental samples**  (mg L^-1^; mg kg^-1^) | | | | | | |
| --- | --- | --- | --- | --- | --- | --- | --- | --- | --- | --- | --- |
|  |  |  |  |  | Cd | Co | Cr | Cu | Ni | Pb | Zn |
| NR | Water | Nag River, Nagpur (MS) | 21.14º N – 79.20º E | NR1-NR9 (9) | 2.52* | 12.1* | 67.6* | 0.05* | 0.04 | 36.99 | 0.16* |
| MF1 | Bulk Soil | Agricultural soil irrigated with Nag river water, Pardi, Nagpur (MS) | 21.14º N – 79.19º E | MF1-MF6 (6) | 11.19 | 67.06 | 67.56 | 0.18 | 94.32 | 217.40 | 0.33 |
| MF2 | Bulk Soil | Nag river irrigated Agricultural Land, Aasoli, Nagpur (MS) | 21.14º N – 79.17º E | MF7-MF10 (4) | 10.76 | 66.40 | 66.56 | 0.19 | 99.20 | 275.40 | 0.38 |
| MF3 | Bulk Soil | Nag river irrigated Agricultural Land, Mahalgaon, Nagpur (MS) | 21.14º N – 79.21º E | MF11- MF12 (2) | 10.62 | 64.54 | 77.20 | 0.13 | 117.56 | 244.00 | 0.31 |
| BA | Bulk Soil | Municipal Sewage Waste dumping site, Bhanpura, Bhopal (MP) | 23.29º N, 77.42º E | BA1-BA8 (8) | 7.47 | 33.20 | 54.98 | 0.16 | 74.62 | 204.00 | 0.31 |
| BB | Bulk Soil | Municipal Sewage Waste dumping site, Bhanpura, Bhopal (MP) | 23.28º N – 78.41º E | BB1-BB16 (16) | 11.48 | 58.98 | 156.92 | 0.79 | 144.00 | 1716.00 | 1.29 |

*Low content in µg L^-1^

**Supplementary Information Table S2:** Minimum inhibitory concentration (MIC) of different heavy metals for potential bacterial isolates

| **Sl. No.** | **Bacterial Isolates** | **MIC in mg L^-1^** | | | | | | | | |
| --- | --- | --- | --- | --- | --- | --- | --- | --- | --- | --- |
|  |  | **Arsenic** | **Cadmium** | **Cobalt** | **Copper** | **Chromium** | **Lead** | **Mercury** | **Nickel** | **Zinc** |
|  | BA3 | 500 | 100 | 70 | - | 120 | 250 | - | 150 | 100 |
|  | BA4 | 500 | 100 | 80 | - | 120 | 250 | - | 150 | - |
|  | BB4 | 250 | 100 | 80 |  | 100 | 200 | - | 100 | - |
|  | BB12 | 300 | 150 | 100 | - | 150 | 300 | - | 150 | - |
|  | BB13 | 600 | 100 | 100 | 200 | - | 200 | - | 150 | - |
|  | BB14 | 600 | 100 | - | 200 | - | 200 | - | - | - |
|  | BB15 | - | 50 | - | - | - | - | - | - | - |
|  | NR5 | 1100 | 100 | 150 | 200 | 500 | 1000 | 100 | 150 | 400 |

**Supplementary Information Table S3:** Cadmium accumulation in bacterial pellet (P) and supernatant (S) of different bacterial isolates (BA3, BA4, BB4, BB12, BB13, BB14, BB15 and NR5 grown in nutrient broth containing 90 mg L^-1^Cd at different time intervals using ICP-OES in biosorption analysis

|  | **Cd concentration in the supernatant (S*) and bacterial pellets (P**)** | | | | | | | | | | | | | | | |
| --- | --- | --- | --- | --- | --- | --- | --- | --- | --- | --- | --- | --- | --- | --- | --- | --- |
| **Time after inoculation** | **BA3** | | **BA4** | | **BB4** | | **BB12** | | **BB13** | | **BB14** | | **BB15** | | **NR-5** | |
|  | S | P | S | P | S | P | S | P | S | P | S | P | S | P | S | P |
| 12 h | 61.02  ±18.09* | 78.87  ±0.64_e_ | 61.78  ±2.34* | 55.33  ±2.42^e^ | 59.44  ±5.32* | 51.97  ±1.57^e^ | 61.58  ±2.99* | 77.53  ±2.25^e^ | 63.82  ±4.94* | 74.33  ±0.71^e^ | 59.42  ±6.42* | 80.87  ±1.63^e^ | 35.94  ±1.43* | 50.03  ±24.04^e^ | 57.57  ±3.11* | 36.85  ±1.46^e^ |
| 24 h | 54.53  ±4.04 | 88.47  ±1.17_d_ | 53.39  ±11.32 | 125.37  ±1.21^d^ | 56.61  ±6.25 | 76.73  ±2.29^d^ | 59.56  ±6.15 | 101.00  ±0.56^d^ | 61.09  ±3.18 | 86.80  ±4.29^d^ | 55.03  ±11.17 | 125.03  ±3.25^d^ | 32.07  ±0.79 | 92.43  ±0.71^d^ | 51.80  ±10.76 | 106.47  ±2.72^d^ |
| 120 h | 50.11  ±1.57 | 132.33  ±3.51_c_ | 49.61  ±9.21 | 147.23  ±2.16^c^ | 49.85  ±7.00 | 166.33  ±0.85^c^ | 52.36  ±10.53 | 146.87  ±3.60^c^ | 51.46  ±13.29 | 116.83  ±2.28^c^ | 58.42  ±3.27 | 175.00  ±1.30^a^ | 27.27  ±5.19 | 238.33  ±1.59^b^ | 49.27  ±14.40 | 136.03  ±2.08^b^ |
| 168 h | 44.95  ±2.80 | 367.63  ±3.27^a^ | 48.53  ±7.90 | 277.00  ±3.61^a^ | 58.11  ±6.66 | 188.10  ±1.85^b^ | 47.97  ±4.03 | 266.33  ±0.76^b^ | 48.87  ±5.72 | 315.50  ±2.29^a^ | 55.33  ±7.25 | 151.67  ±1.89^c^ | 24.78  ±3.37 | 252.30  ±11.27^a^ | 48.40  ±7.66 | 126.60  ±1.87^c^ |
| 240 h | 40.07  ±17.59 | 355.33  ±3.06^b^ | 44.22  ±11.63 | 214.93  ±3.30^b^ | 48.24  ±8.93 | 207.43  ±2.87^a^ | 39.43  ±13.55 | 435.00  ±1.00^a^ | 45.40  ±4.94 | 133.50  ±2.78^b^ | 52.71  ±11.76 | 152.40  ±1.73^b^ | 19.62  ±3.32 | 204.20  ±4.85^c^ | 44.49  ±9.03 | 305.13  ±4.72^a^ |

Data are mean of three replicates with SD (±); *mg L^-1^, ** mgkg^-1^

#Data analyzed by Duncan’s multiple range test (DMRT), values within the same column with different letters are significantly different (p ≤ 0.05) by ANOVA; values are means ± SD (n = 3); SD = standard deviation. ‘a’ stands for highest variation, followed by ‘b’ and lowest by ‘c’ from the experimental control. No significant difference was observed in supernatant, although the highest significance among five values at different time interval marked as asterisk*

**Supplementary Information Table S4:** Antibiotic profiling of *Ochrobactrum intermedium* BB12

| S.No. | Name of Antibiotic | Quantity (µg) | Symbol | Observed Clear Zone around the colony (mm) |
| --- | --- | --- | --- | --- |
|  | Amikacin | 30 | AK | 28 |
|  | Ampicillin | 10 | AMP | - |
|  | Amoxycillin | 10 | AMX | - |
|  | Cefaperazone | 75 | CPZ | 11 |
|  | Cefadroxil | 30 | CFR | - |
|  | Ceftazidime | 30 | CAZ | - |
|  | Ceftriaxone | 30 | CTR | - |
|  | Chloramphenial | 30 | C | 22 |
|  | Ciprofloxacin | 5 | CIP | 23 |
|  | Cloxacillin | 1 | COX | - |
|  | Co-Trimoxazole | 25 | COT | 16 |
|  | Erythromycin | 15 | E | 19 |
|  | Gentamicin | 10 | GEN | 26 |
|  | Nalidixic Acid | 10 | NA | 24 |
|  | Netillin | 10 | NET | 31 |
|  | Nitrofurantoin | 300 | NIT | - |
|  | Norfloxacin | 10 | NX | 26 |
|  | Penicillin | 10 | P | - |
|  | Tobramycin | 10 | TOB | 27 |
|  | Vancomycin | 30 | VA | - |

**Supplementary Information Table S5:** Plant growth promoting attributes of Cd tolerant bacterium *Ochrobactrum intermedium* BB12

| **PGP Traits** | **Values** |
| --- | --- |
| Siderophore production (colony diameter) | 7.8±0.78mm |
| Ammonia production | Negative |
| Phosphate solubilization (soluble P) | 263.83±8.38µg mL^−1^ |
| Indole-3-acetic acid production | 48.85±1.68µg mL^−1^ |
| Potassium solubilization index | 1.63±0.081 |

# Supplementary Information Table S6: Principal Components Analysis (Eigenvalues and Percentage of Variance) of the impact of different concentration of Cd on plant growth promoting attributes of BB12

| Principal Components | Eigenvalue | % Variance | Eig 2.5% | Eig 97.5% |
| --- | --- | --- | --- | --- |
| 1 | 1081.79 | 99.645 | 99.46 | 100 |
| 2 | 3.84561 | 0.35422 | 2.0776E-31 | 0.54043 |
| 3 | 0.00510039 | 0.0004698 | 5.0063E-66 | 0.0004698 |

# Supplementary Information Table S7: Principal Components Analysis of the impact of BB12 on Cd uptake reduction and physiological growth parameters at different concentrations

| Principal Components | Eigenvalue | % Variance | Eig 2.5% | Eig 97.5% |
| --- | --- | --- | --- | --- |
| 1 | 5104.33 | 90.976 | 5.74E-63 | 99.673 |
| 2 | 495.411 | 8.8299 | 0.28059 | 99.437 |
| 3 | 6.53241 | 0.11643 | 2.48E-68 | 0.34002 |
| 4 | 3.60923 | 0.064329 | 1.24E-98 | 0.14701 |
| 5 | 0.739706 | 0.013184 | 1.67E-64 | 0.018717 |

**Supplementary Information Table S8:** Effect of the application of *Ochrobactrum intermedium* BB12 on biochemical profile of spinach plants grown in the soil containing 25, 50 and 75 mg kg^-1^of cadmium at 45 and 75 days after sowing (DAS)

| **Physiological parameter** | **DAS** | **Treatments** | | | | | | |
| --- | --- | --- | --- | --- | --- | --- | --- | --- |
|  |  | **T1** | **T2** | **T3** | **T4** | **T5** | **T6** | **T7** |
| Chlorophyll-a (µg g^-1^) | 45 | 29.06±1.78^a^ | 25.62±2.08^ab^ | 25.79±1.93^ab^ | 21.60±2.02^c^ | 25.12±2.15^b^ | 14.14±1.77^d^ | 25.94±0.96^ab^ |
| Chlorophyll-a (µg g^-1^) | 75 | 29.31±0.69^ab^ | 27.99±1.12^b^ | 29.01±1.34^ab^ | 28.45±1.10^b^ | 30.17±1.21^ab^ | 27.61±1.90^b^ | 31.50±1.72^a^ |
| Chlorophyll-b (µg g^-1^) | 45 | 43.73±4.06^c^ | 38.75±2.94^cd^ | 54.35±3.78^ab^ | 36.64±2.98^d^ | 49.95±2.46^b^ | 39.59±1.42^cd^ | 58.86±4.61^a^ |
| Chlorophyll-b (µg g^-1^) | 75 | 60.35±3.68^a^ | 45.08±2.54^b^ | 48.19±3.31^b^ | 61.49±2.45^a^ | 63.09±5.76^a^ | 42.96±1.50^b^ | 47.50±2.36^b^ |
| Total Chlorophyll (µg g^-1^) | 45 | 70.53±6.24^b^ | 68.91±4.68^b^ | 81.57±5.81^a^ | 59.24±5.09^c^ | 76.39±4.67^ab^ | 66.63±2.42^bc^ | 74.44±6.49^ab^ |
| Total Chlorophyll (µg g^-1^) | 75 | 88.31±4.57^a^ | 74.36±3.16^bc^ | 77.17±4.43^bc^ | 89.91±3.55^a^ | 93.23±4.97^a^ | 70.54±2.91c | 78.97±3.53^b^ |
| Proline (µmoleg^-1^ FW)  Proline (µmoleg^-1^ FW) | 45 | 0.63±0.04^c^ | 01.44±0.27^b^ | 0.76±0.02^c^ | 1.80±0.05_a_ | 0.92±0.04^c^ | 1.89±0.27^a^ | 1.09±0.23^c^ |
|  | 75 | 0.53±0.07^d^ | 1.30±0.18^b^ | 0.72±0.04^cd^ | 1.55±0.05^ab^ | 0.85±0.07^c^ | 1.67±0.20^a^ | 0.54±0.05^d^ |
| SOD (U activity g^-1^ FW)  SOD (U activity g^-1^ FW) | 45 | 0.07±0.02^a^ | 0.20±0.01^c^ | 0.16±0.01^d^ | 0.27±0.01^a^ | 0.18±0.01^b^ | 0.29±0.02^ab^ | 0.25±0.01^cd^ |
|  | 75 | 0.09±0.02^e^ | 0.10±0.01^b^ | 0.09±0.01^c^ | 0.10±0.01^a^ | 0.07±0.01^bc^ | 0.11±0.02^bc^ | 0.09±0.01^d^ |

#T1: control without Cd and BB-12; T2, T4 and T6: metal control at 25, 50 and 75 mg kg^-1^ Cd respectively without BB-12; T3, T5 and T7: BB-12 amended plants under Cd (25, 50 and 75 mg kg^-1^ Cd, respectively) content

#Data analyzed by Duncan’s multiple range test (DMRT), values within the same column with different letters are significantly different (p ≤ 0.05) by ANOVA; values are means ± SD (n = 3); SD = standard deviation. ‘a’ stands for highest variation, followed by ‘b’ and lowest by ‘d’ from the experimental control
